# Mitochondrial Ca^2+^ oscillation induces mitophagy initiation through the PINK1-Parkin pathway

**DOI:** 10.1038/s41419-021-03913-3

**Published:** 2021-06-19

**Authors:** Zhengying Yu, Haipeng Wang, Wanyi Tang, Shaoyang Wang, Xiaoying Tian, Yujie Zhu, Hao He

**Affiliations:** 1 School of Biomedical Engineering, Shanghai Jiao Tong University, Shanghai, China; 2 Department of Dermatology, Shanghai Ninth People’s Hospital, School of Medicine, Shanghai Jiao Tong University, Shanghai, China

**Keywords:** Mitophagy, Calcium signalling

## Abstract

Dysregulation of the PINK1/Parkin-mediated mitophagy is essential to Parkinson’s disease. Although important progress has been made in previous researches, the biochemical reagents that induce global and significant mitochondrial damage may still hinder deeper insights into the mechanisms of mitophagy. The origin of PINK1/Parkin pathway activation in mitophagy remains elusive. In this study, we develop an optical method, ultra-precise laser stimulation (UPLaS) that delivers a precise and noninvasive stimulation onto a submicron region in a single mitochondrial tubular structure. UPLaS excites localized mitochondrial Ca^2+^ (mitoCa^2+^) oscillations with tiny perturbation to mitochondrial membrane potential (MMP) or mitochondrial reactive oxygen species. The UPLaS-induced mitoCa^2+^ oscillations can directly induce PINK1 accumulation and Parkin recruitment on mitochondria. The Parkin recruitment by UPLaS requires PINK1. Our results provide a precise and noninvasive technology for research on mitophagy, which stimulates target mitochondria with little damage, and reveal mitoCa^2+^ oscillation directly initiates the PINK1-Parkin pathway for mitophagy without MMP depolarization.

## Introduction

Parkinson’s disease (PD), the second most common neurodegenerative disorder, is consistently associated with mutations of Parkin and PTEN­induced kinase1 (PINK1) genes [1, 2]. These mutations affect many mitochondrial functions and have secondary effects on oxidative phosphorylation and other processes [3, 4]. Mitophagy has been found regulated by the activity of Parkin and PINK1 and usually becomes abnormal in PD [5, 6]. Specifically, dysregulation of mitophagy by PINK1-Parkin is believed as one of the main factors contributing to cell death and pathogenesis in PD [7–9]. The mechanism of mitophagy and its dysregulation is essential to better understandings and insights into PD.

In the PINK1/Parkin mediated mitophagy, PINK1 localizes on the mitochondrial outer membrane, phosphorylates ubiquitin molecules there, and recruits Parkin that originally resides in the cytosol to the damaged mitochondria. Parkin further governs the developing LC3 isolation membrane for the formation of autophagosomes that are delivered to the lysosome for degradation [7, 10–12]. The activation of the PINK1-Parkin pathway is generally believed rooted in the loss of mitochondrial membrane potential (MMP), one of many physiological responses of the mitochondria to stress and inevitable subsequences of mitochondrial damage [13]. The accumulation of PINK1 on the outer mitochondrial membrane can then be induced by the failure of importing and degrading PINK1 in dysfunctional mitochondria or some other molecular signalings [14, 15]. PINK1 stabilizes there and recruits Parkin to initiate mitophagy.

In previous researches, potent chemical reagents like carbonyl cyanide 3­chlorophenylhydrazone (CCCP) are extensively used to produce stress to mitochondria, resulting in rapid, significant, and permanent MMP depolarization [13, 16–18]. Notably, unfolded protein expressed in the mitochondrial matrix and proteotoxic stress also activates mitophagy through this pathway but without loss of MMP [19, 20]. It was reported that PINK1 regulates calcium efflux from mitochondria [21]. The PINK1 deficiency causes accumulation and overload of mitochondrial calcium [22]. In this regard, the possibility of perturbed mitochondrial Ca^2+^ signals as causes or consequences of mitophagy induction was also proposed [23]. However, the activation origin of the PINK1-Parkin pathway remains elusive.

In this study, we developed a noninvasive ultra-precise laser stimulation (UPLaS) technology to individual mitochondria that excites mitochondrial Ca^2+^ (mitoCa^2+^) oscillations with tiny perturbation to MMP or mitochondrial reactive oxygen species (mitoROS). The mitoCa^2+^ oscillation by UPLaS initiates the PINK1/Parkin pathway for mitophagy. Our results suggest mitoCa^2+^ oscillations as a candidate for the PINK1/Parkin mediated mitophagy initiation.

## Results

### UPLaS excites mitoCa^2+^ oscillations to initiate PINK1/Parkin pathway for mitophagy

**Fig. 1:**
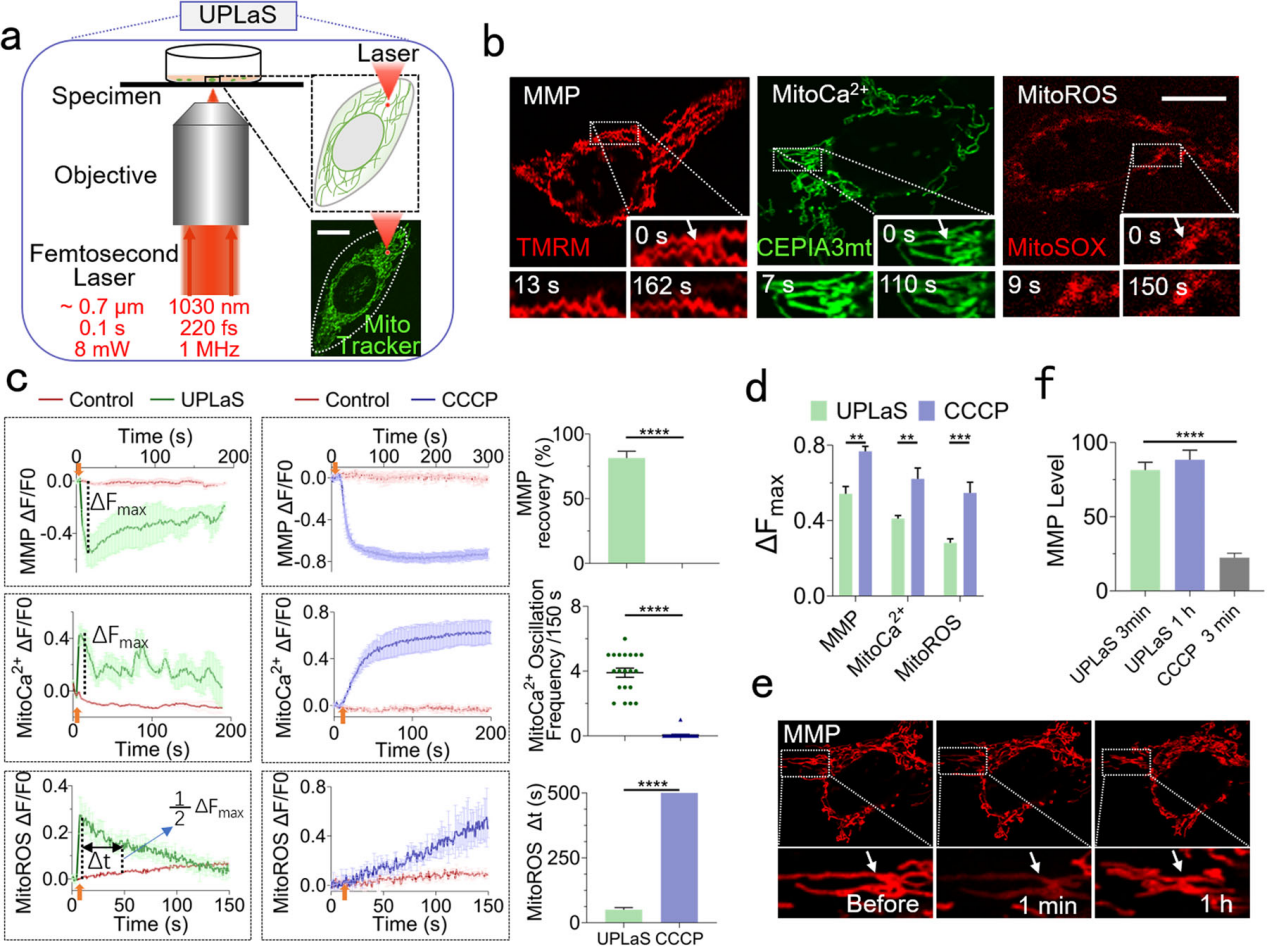
UPLaS excites mitoCa^2+^ oscillations in the target mitochondria with little perturbation to MMP or mitoROS. **a** The setup of UPLaS based on a microscopic system to focus a femtosecond laser onto a diffraction-limit spot on a target single-mitochondrial tubular structure. The laser is controlled by a shutter. **b** The individual mitochondrion stimulated by UPLaS. The responses of MMP, mitoCa^2+^, and mitoROS to UPLaS (at *t* = 5 s) are, respectively, indicated by TMRM, CEPIA3mt, MitoSOX. Inserts: the mitochondrial responses to UPLaS at different time slots. **c** The MMP (*n* = 25), mitoCa^2+^ (*n* = 20), and mitoROS (*n* = 35) level of the mitochondria treated with UPLaS (at *t* = 5 s) and CCCP (since *t* = 10 s, 10 μM), respectively, (left panel). Right panel: the quantified ratio of recovered MMP level over the original at 200 s, mitoCa^2+^ oscillation frequency, and the mitoROS recovery time (Δ*t*) defined by the width at the half maximum of the mitoROS curve, in the mitochondria treated with UPLaS. The Δ*t* by CCCP was defined as 500 s due to its continuous rise in 500 s. Arrows: the UPLaS or CCCP treatment events. **d** The maximum fluorescence amplitude of the MMP, mitoCa^2+^, and mitoROS level of the mitochondria treated with UPLaS and CCCP, respectively. **e** The MMP level before, one minute, and one hour after UPLaS. **f** The quantified MMP levels by UPLaS and CCCP, respectively. Bar: 20 μm. *p < 0.05, **p < 0.01, and ***p < 0.001.

We established the UPLaS method to evoke active localized mitoCa^2+^ oscillations in a single mitochondrial tubular structure, with transient, moderate, and fast-recovered MMP depolarization and mitoROS increase there, without any perturbation to its neighbors. The UPLaS system design is shown in Fig. 1a. A femtosecond laser was tightly focused by a ×60 objective (water immersed, N.A. = 1.2) to a diffraction-limit spot (diameter ~700 nm). The wavelength was at 1030 nm. Such low single-photon energy could hardly generate any photochemical effect. The pulse width at 220 fs and repetition rate of 1 MHz guaranteed little thermal accumulation. In this way, the femtosecond-laser stimulation penetrated through cells with few side effects. In this study, the UPLaS spot was localized onto a single mitochondrial tubular structure and temporally controlled for a 100-ms laser illumination at 8 mW by a shutter. The cells were observed by confocal microscopy continuously to provide real-time mitochondrial dynamics. The UPLaS was performed for a single time in one cell.

We performed UPLaS in SH-SY5Y cells to demonstrate the validity of this method. As shown in Fig. 1b, this technique enables spatiotemporal-specific stimulations to the target individual mitochondria. The MMP of the stimulated mitochondria was transiently depolarized but soon recovered (Fig. 1c, UPLaS at *t* = 5 s). The mitoROS level that can mirror mitochondrial damage increased after UPLaS and returned to the normal level also in a short time (<150 s). Notably, active mitoCa^2+^ oscillations were excited (~4 Ca^2+^ spikes/ 150 s) and lasted for more than 200 s (Fig. 1c). Those results suggest UPLaS could evoke mitoCa^2+^ oscillations with little damage. As a comparison, the cells treated with the classic drug, CCCP (10 μM, since *t* = 10 s) presented destructive consequences immediately (Fig. 1c). The MMP exhibited significant depolarization and could not recover again. The maximum level of MMP depolarization induced by CCCP was significantly higher than it by UPLaS (Fig. 1d). The mitoCa^2+^ and mitoROS both increased continuously and dramatically (and did not fall back in 500 s, Fig. 1c). The maximum mitoCa^2+^ and mitoROS level by CCCP were also significantly higher (Fig. 1d). Particularly, we monitored the MMP level for one hour after UPLaS and found it recovered in 3 min (>80% of the original level) and maintained the healthy state in the following one hour (Fig. 1e, f). Those data suggest UPLaS as a precise and nondestructive stimulation to individual mitochondria.

**Fig. 2:**
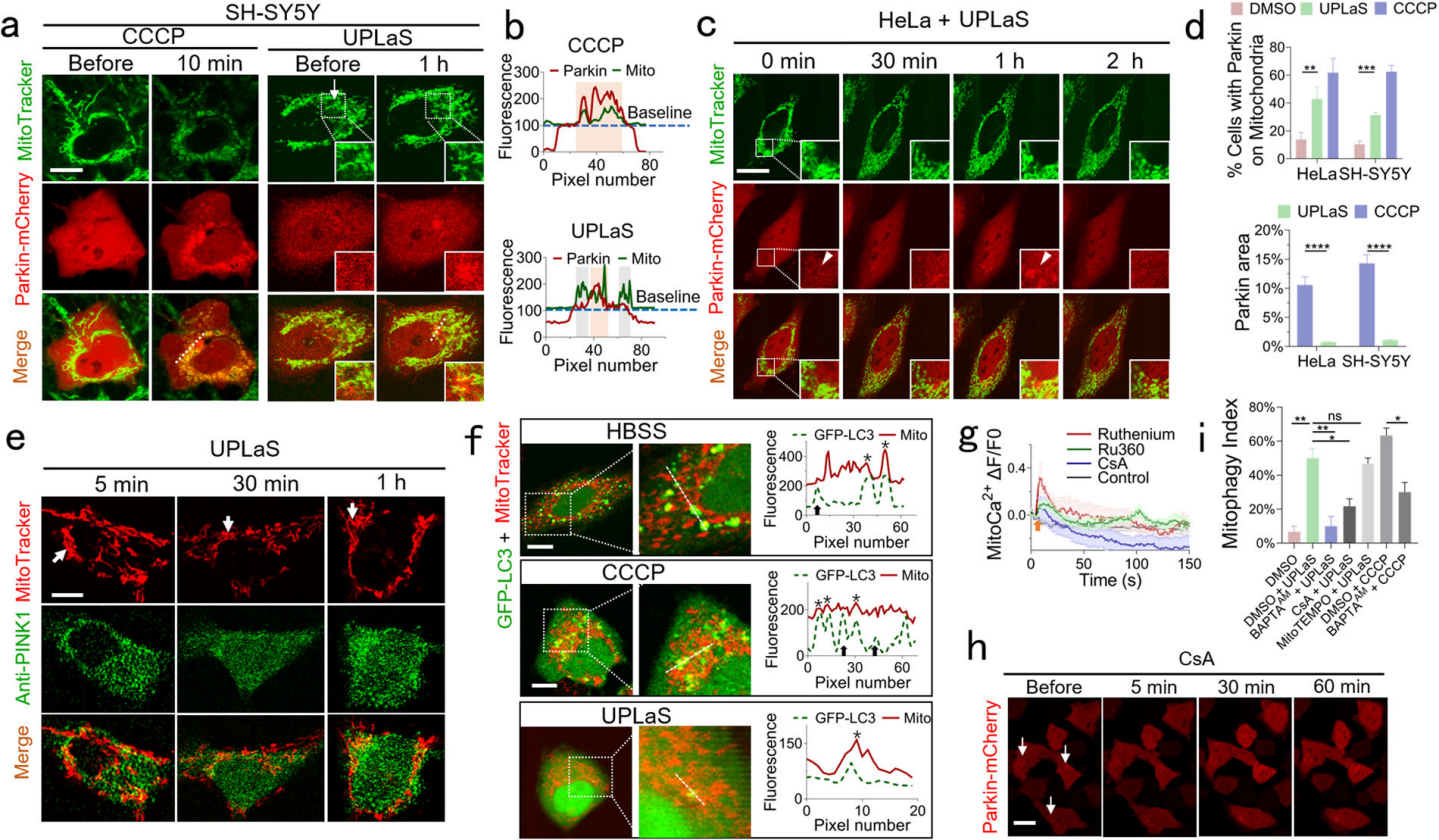
Parkin recruitment and PINK1 accumulation by UPLaS. **a** SH-SY5Y cells transfected with Parkin-mCherry (red) and stained with MitoTracker Green (green). Arrow: the UPLaS location. Inserts: Parkin recruitment to mitochondria with UPLaS. **b** The fluorescence intensity plot along a randomly selected line in cells in **a**. **c** The time-lapse dynamics of Parkin-mCherry after UPLaS. **d** The cell ratio of Parkin recruitment in HeLa (*n* = 20) and SH-SY5Y (*n* = 35) cells, respectively up and the Parkin recruitment area ratio over the whole cells down. **e** The PINK1 localization after UPLaS. **f**. The colocalization of mitochondria (MitoTracker Red) and autophagosomes (GFP-LC3) 1.5 h after UPLaS. Note in the HBSS and CCCP groups, the LC3 puncta coincided with mitochondria (stars) or misaligned with mitochondria (arrows). **g** The mitoCa^2+^ after UPLaS in the presence of Ru360 (10 μM, *n* = 20), ruthenium (10 μM, *n* = 20), and CsA (10 μM, *n* = 20), respectively. **h** The Parkin-mCherry dynamics in cells treated with CsA after UPLaS (*n* = 25). **i** The mitophagy index in cells treated with UPLaS in the presence of CsA, BAPTA-AM, and MitoTEMPO, respectively, or treated with CCCP in the presence of BAPTA-AM (*n* = 30 in each group). Bar: 20 μm. *p < 0.05, **p < 0.01, ***p < 0.001, ****p < 0.0001.

We then demonstrated UPLaS induced Parkin recruitment on the stimulated mitochondria. Parkin and mitochondria in SH-SY5Y cells were visualized simultaneously by Parkin-mCherry and MitoTracker Green, respectively, for the examination of colocalization of them. As shown in Fig. 2a, b, Parkin-mCherry aggregated only in the mitochondria with UPLaS one hour later, indicating localized Parkin recruitment there. The cells with CCCP treatment (since *t* = 10 s) exhibited global Parkin recruitment in the whole cell. UPLaS also worked well in HeLa cells (Fig. 2c). The time-lapse dynamics of Parkin-mCherry indicated the most significant Parkin recruitment to the stimulated mitochondria was at one hour after UPLaS. We quantified the efficiency of UPLaS-induced Parkin recruitment by calculating the ratio of cells exhibiting Parkin recruitment in the UPLaS-mitochondria over the total treated cells within an hour. The efficiency was around 40% in HeLa and 32% in SH-SY5Y cells, respectively (Fig. 2d). The Parkin recruitment area was highly confined in the UPLaS location. The efficiency of CCCP-induced Parkin recruitment was around 60%.

Then we accessed the possibility of UPLaS initiated mitophagy. At first, we measured the colocalization of PINK1 (visualized by immunofluorescence (IF) microscopy) and mitochondria (indicated by MitoTracker Red). The IF microscopy of PINK1 was performed at 5, 30, and 60 min after UPLaS. As shown in Fig. 2e, the level of PINK1 on the stimulated mitochondria could be found higher one hour after UPLaS. Then we examined the colocalization of autophagosome (indicated by GFP-LC3) and mitochondria. As a positive control, cells were incubated in HBSS for the starvation that caused both mitophagy and autophagy, in which the autophagosomes coincided or misaligned with mitochondria (Fig. 2f). The combination of autophagosomes and lysosomes was further confirmed in Supplementary Fig. 1. Some autophagosomes in the cells treated by CCCP did not colocalize with mitochondria either. In contrast, the mitochondria with UPLaS all presented consistent coincidence with autophagosomes (Fig. 2f). Those data suggest the PINK1/Parkin pathway was initiated in the mitochondria with UPLaS for mitophagy.

Considering MMP or mitoROS both recovered back to normal in a very short time (100–200 s) and maintained healthy in the following one hour after UPLaS, we suspected the unique mitoCa^2+^ oscillation by UPLaS took the responsibility for the mitophagy initiation. To verify this hypothesis, we blocked the Ca^2+^ channels of mitochondria by using Ru360 (10 μM, 30 min), a specific inhibitor of the mitochondrial calcium uniporter (MCU), Ruthenium (10 μM, 30 min), a specific inhibitor of the voltage-dependent anion channel (VDAC) in the outer mitochondrial membrane, and Cyclosporine A (CsA, 1 μM, 30 min), the inhibitor of mitochondrial permeability transition pores (mPTPs), to treat cells before UPLaS, respectively. In the presence of Ru360 or Ruthenium, the mitoCa^2+^ oscillation by UPLaS was significantly suppressed, as shown in Fig. 2g. Particularly, the inhibition of mPTPs by CsA could even totally inhibit the mitoCa^2+^ oscillation. The mitochondria did not show any Parkin recruitment after UPLaS in cells treated with CsA (Fig. 2h). We further examined the ratio of cells with coincident colocalization of mitochondria and autophagosomes over the total cells with UPLaS, by defining it as the mitophagy index. It could be found in Fig. 2i that this index by UPLaS with CsA was then significantly lower than it of control (with DMSO). We finally used BAPTA-AM to chelate intracellular Ca^2+^ before UPLaS. The mitophagy index without Ca^2+^ after UPLaS also decreased significantly. The Ca^2+^ chelation could even significantly reduce the mitophagy index by CCCP. To exclude the influence of the transient mitoROS generation by UPLaS, we treated cells with mitoTEMPO to scavenge the mitoROS. Without mitoROS, the mitophagy index was not influenced (Fig. 2i), indicating the transient mitoROS by UPLaS did not contribute to the mitophagy initiation. Taken together, the mitoCa^2+^ oscillation by UPLaS plays an essential role in mitophagy initiation.

### PINK1 is indispensable in the mitoCa^2+^ oscillation-triggered mitophagy initiation

**Fig. 3:**
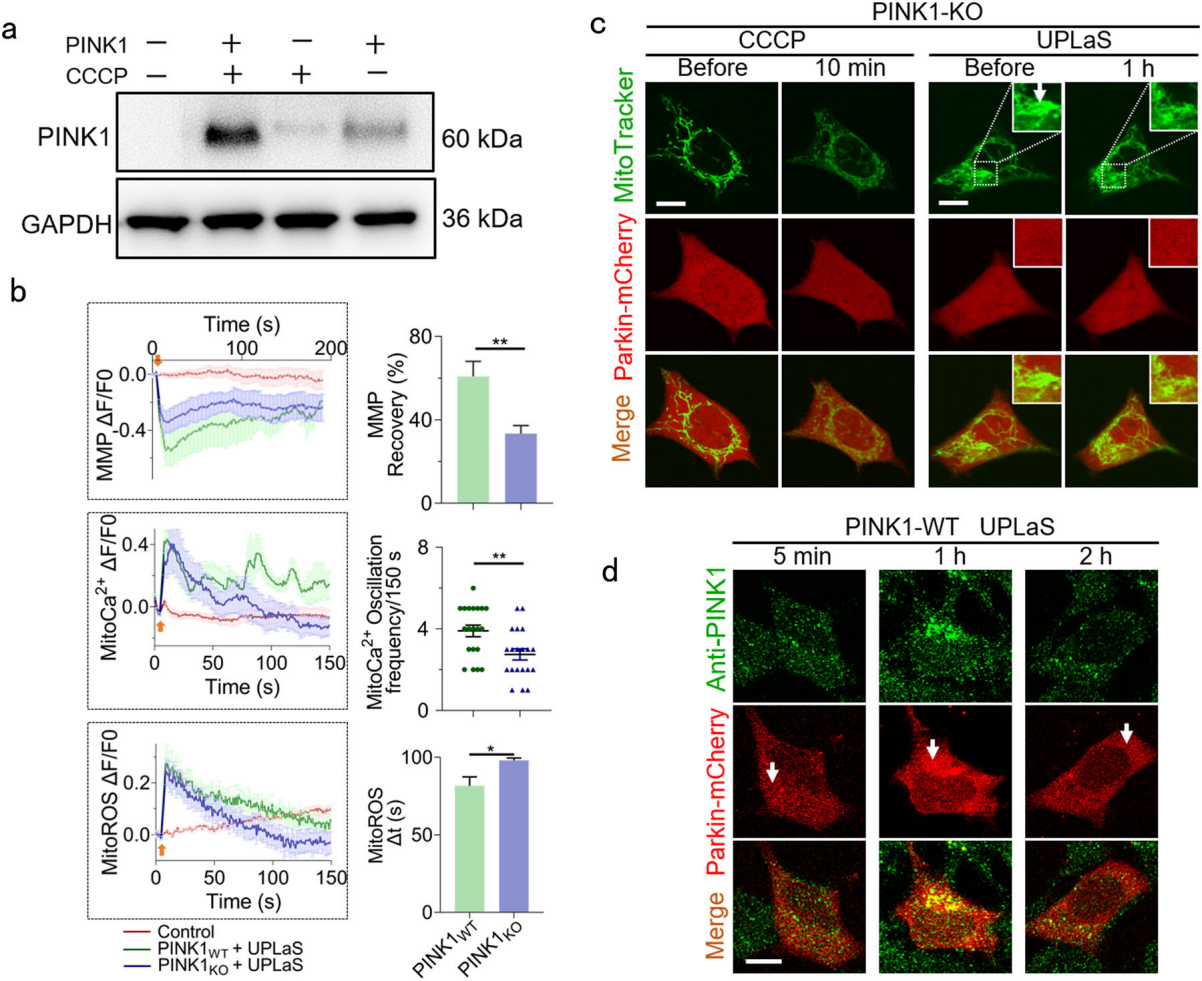
UPLaS could not recruit Parkin in PINK1-KO SH-SY5Y cells. **a** The Western Blot of PINK1 in PINK1-KO and wild-type SH-SY5Y cells with or without CCCP (for 1 h), respectively. **b** The MMP (*n* = 25), mitoCa^2+^ (*n* = 20), and mitoROS (*n* = 35) of the target mitochondria in the PINK1-KO cells after UPLaS. **c** The Parkin recruitment (Parkin-mCherry) to mitochondria (MitoTracker Green) treated with CCCP and UPLaS. **d** The IF microscopy of PINK1 and Parkin in WT cells after UPLaS. Bar: 20 μm. *p < 0.05, **p < 0.01.

We finally presented PINK1 is indispensable in such a mitoCa^2+^ oscillation-triggered mitophagy initiation. Firstly, we established the PINK1-knockout (KO) SH-SY5Y cell line by CRISPR. The PINK1 level was measured by Western Blot (Fig. 3a) and the DNA was sequenced as Supplementary Fig. 2. The CCCP treatment could upregulate the PINK1 level in the wild-type (WT) cells but hardly in the PINK1-KO cells (Fig. 3a). In the PINK1-KO cells, the MMP recovery and mitoCa^2+^ oscillation after UPLaS were significantly suppressed (Fig. 3b). The mitoCa^2+^ oscillation frequency excited by UPLaS in PINK1-KO cells decreased, but still remained around ~3 spikes/150 s. Under this condition, the UPLaS or CCCP could not recruit any Parkin to mitochondria in the PINK1-KO cells (Fig. 3c). But in the WT cells, UPLaS could induce aggregation of Parkin and consistent colocalization of PINK1 and Parkin (Fig. 3d). Those results suggest PINK1 is necessary to Parkin recruitment by the UPLaS-excited mitoCa^2+^ oscillations.

**Fig. 4:**
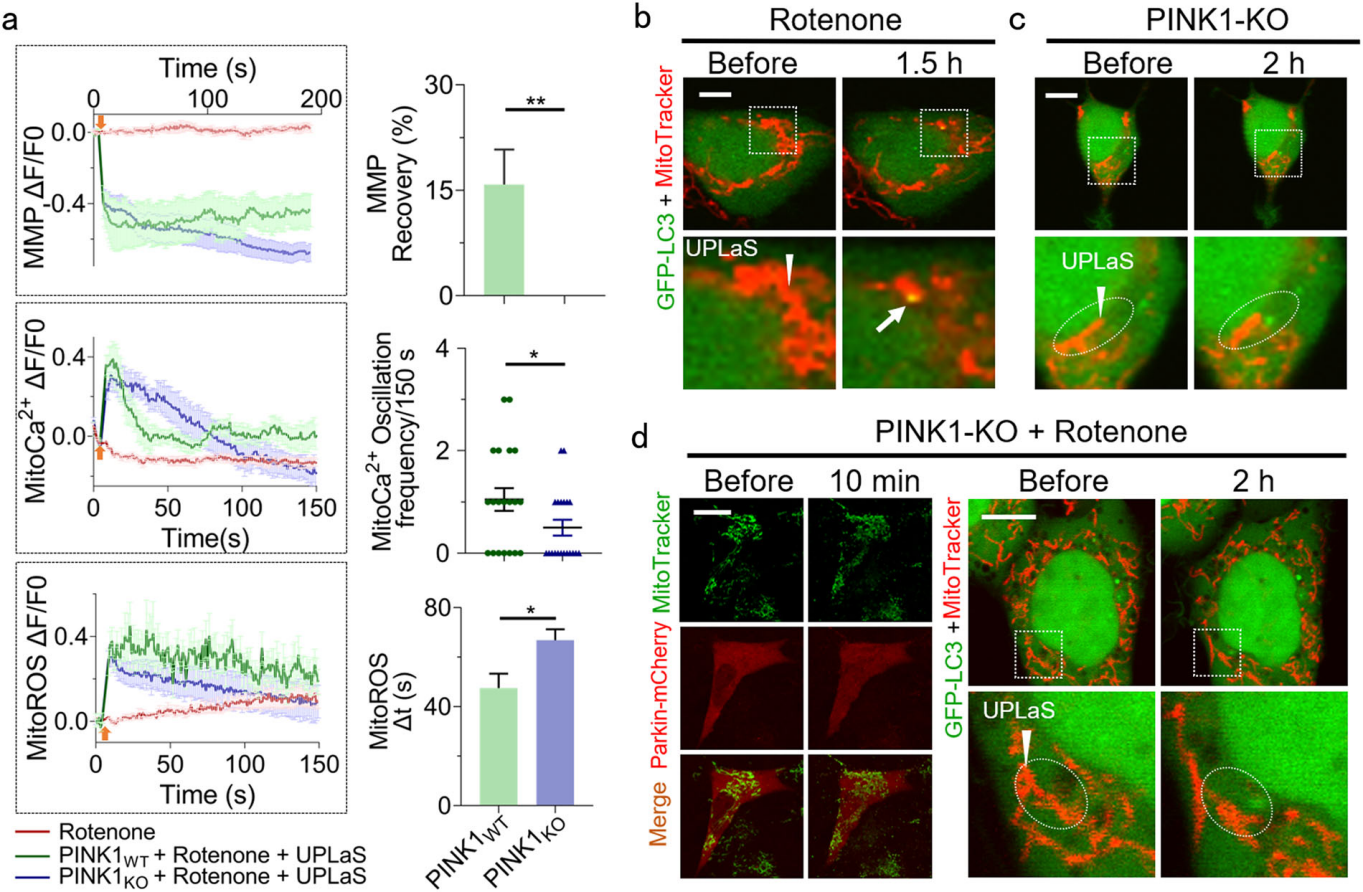
UPLaS-induced mitophagy in cells treated with rotenone. **a** The MMP (*n* = 25), mitoCa^2+^ (*n* = 20), and mitoROS (*n* = 35) of the rotenone-treated cells with or without PINK1 after UPLaS. **b** Colocalization of mitochondria and LC3 puncta in PINK-WT cells treated with rotenone (*n* = 27). **c** No colocalization of mitochondria and LC3 puncta could be found in PINK1-KO cells (*n* = 24). **d** No Parkin recruitment or autophagosome colocalization with mitochondria found in rotenone-treated PINK1-KO cells (*n* = 30). Arrowheads: the UPLaS location. Arrow: mitophagy. Bar: 20 μm. *p < 0.05, **p < 0.01.

Secondly, we investigated if the suppressed mitoCa^2+^ oscillation could still activate mitophagy. The WT SH-SY5Y cells were treated with rotenone to induce mitochondrial malfunction, which is a widely-used cell model of Parkinson’s disease [24–27]. Under this condition, the UPLaS-excited mitoCa^2+^ oscillations were suppressed to ~1 spikes/150 s (Fig. 4a). Notably, the depolarized MMP and increasing mitoROS could hardly recover to the normal level as shown in Fig. 4a and Supplementary Fig. 3. The mitophagy index by UPLaS in those rotenone-treated PINK1-WT SH-SY5Y cells could achieve ~20 ± 6.67% (Fig. 4b), significantly lower than it without rotenone treatment (~50 ± 8.16% in Fig. 2i). The weak mitoCa^2+^ oscillations hindered the mitophagy initiation in those rotenone-treated cells, although their MMP was depolarized.

In contrast, in PINK1-KO SH-SY5Y cells that could be excited with moderate mitoCa^2+^ oscillations by UPLaS (Fig. 3b), no autophagosomes could be found in the UPLaS location as shown in Fig. 4c, although the MMP was partially depolarized. As a control, to rule out the possibility that rotenone treatment directly promoted mitophagy without PINK1, the PINK1-KO cells were treated with rotenone. The damaged mitochondria exhibited even fewer mitoCa^2+^ oscillations and less MMP recovery by UPLaS (Fig. 4a). Under this condition, no Parkin recruitment or colocalization of the autophagosome with mitochondria could be found (Fig. 4d). Hence the UPLaS-induced mitophagy in rotenone-treated cells was still PINK1-mediated. Taken together, mitoCa^2+^ oscillation initiates mitophagy through the PINK1-Parkin pathway.

## Discussion

In previous works, PINK1/Parkin mediated mitophagy has been well studied, in which CCCP was extensively used to depolarize mitochondria and trigger mitophagy. Notably, CCCP is a toxic drug, induces complete depolarization of MMP, and generates significant high-level mitoROS. With CCCP treatment, the mitochondria and cells undergo a very abnormal state that can rarely be encountered under physiological conditions. The toxicity may further lead to global damages to the mitochondria in the whole cell and even apoptosis or necrosis. Those physiological changes may affect the investigations of mitophagy molecular pathways [28, 29]. The UPLaS method developed in this study exhibits a very protective and precise stimulation to single mitochondria with tiny perturbation to MMP and mitoROS. The MMP depolarization and mitoROS increase recover to normal level in a very short time (~100–200 s).

Mitochondrial Ca^2+^ signaling has been noticed related to mitophagy but remains obscure [23]. By UPLaS, we demonstrate the mitoCa^2+^ oscillation can initiate the PINK1-Parkin pathway for mitophagy. Rotenone-treated SH-SY5Y cells have been widely used as a model of PD disease. As shown in Fig. 4a, the UPLaS-induced mitoCa^2+^ oscillation in rotenone-treated SH-SY5Y cells was suppressed to a single-time Ca^2+^ spike, the mitophagy index then decreased significantly (Fig. 4b). It could be suspected that the reason why mitophagy is abnormal in PD might be the suppressed mitoCa^2+^ dynamics as in the rotenone-treated SH-SY5Y cells.

In the PINK1-KO cells, the moderate mitoCa^2+^ oscillations could not initiate the PINK1-Parkin pathway. This result is consistent with the view that PINK1 detects mitochondrial damage and mitoCa^2+^. According to our data, PINK1 is quite sensitive to mitoCa^2+^ oscillations to recruit Parkin and activate mitophagy. The kinase domain of PINK1, homologous to the Calcium/Calmodulin-dependent kinase family of the kinase [30], may be activated by the UPLaS-excited mitoCa^2+^ oscillations. Moreover, the molecular targets that Parkin ubiquitinates on the outer mitochondrial membrane including MFNs [31], DNM1L [32], VDAC1 [33], and BCL-2 [34], are all mitoCa^2+^ sensors. Specifically, the autophagosomes in the UPLaS location close to the target mitochondria rather than coincident with it, (Fig. 4c, d) in PINK1-KO cells, might be the subsequent phenomena of mitochondria clearance after mitophagy according to the previous work [33].

## Materials and methods

### Cell culture and transfection

HeLa cells were cultured in Dulbecco’s Modified Eagle’s Medium (DMEM, Hyclone) supplemented with 10% fetal bovine serum (FBS, Gibco), 1% penicillin/streptomycin (Gibco). Neuroblastoma SH‑SY5Y cells were grown in DMEM/HAM’s F12 (Gibco) supplemented with 10% fetal bovine serum (FBS, Gibco), 1% penicillin/streptomycin (Gibco), 1% GlutaMAX™ (Gibco), 1% non-essential amino acids, and 1% Sodium Pyruvate (Gibco). All cells were incubated in 35-mm dishes (Corning) at 37 °C in a humidified 5% CO_2_ atmosphere. First, cells (~5 × 10^5^ cells/mL) were seeded in a 35-mm confocal dish for 24 h, which enabled cells to reach 60 to 80% confluent at the time of the transfection. Then, cells were transiently transfected with 1 μg plasmid for 4–5 h using jetPRIME (Polyplus) according to manufacturer’s instructions and incubated with refresh culture medium for 24–48 h.

### Fluorescent microscopy of MMP, mitoCa^2+^, and mitoROS

Fluorescent probes Tetramethylrhodamine, Methyl Ester, Perchlorate (TMRM, T668, Invitrogen), MitoSOX^TM^ (M36008, Invitrogen), and plasmid CEPIA3mt (a generous gift from Dr. Franck Polleux) were used, respectively, for indicating MMP, mitoROS, and mitoCa^2+^ in live cells at the real-time. Cells were transfected with the plasmid CEPIA3mt for 24–48 h at 37 °C before experiments. TMRM and MitoSOX were used to stain cells before experiments according to the protocol from the manufacturers. After staining with dyes or transfected with plasmids, cells were washed with 3× PBS and the buffer was replaced with a fresh culture medium and protected from ambient light during incubation.

For confocal microscopy, all green dyes or fluorescent proteins, including GFP, CEPIA3mt, MitoTracker Green, and GFP-LC3, were excited by a laser at 473 nm at around 0.1 mW, and the red ones, including TMRM, MitoSOX, MitoTracker Red, Parkin-mCherry were excited by a 543 nm laser at about 0.1 mW. The green fluorescence was detected with a band filter (490–525 nm) and the red with (560–620 nm). Images were acquired at a speed of 2.2 μs for one pixel in a frame (512 × 512 pixels) or (1024 × 1024 pixels).

### The UPLaS scheme

The Optical system was established on confocal microscopy (FV1200, Olympus) coupled with a femtosecond laser (BlueCut, Menlo, 1030 nm, 1 MHz, 220 fs). The laser was focused by an objective (water-immersed, ×60, 1.2 N.A.). Experiments were performed at 37 °C controlled by a mini-incubator on the microscope stage. Cells were localized by Petri dishes with a grid at the bottom (Grid-500, Ibidi).

The femtosecond-laser stimulation was delivered by the galvo-mirrors to the predefined region of the targeted mitochondrion, as demonstrated in Fig. 1a. The laser exposure was controlled by a mechanical shutter that set for 0.1 s open, which was synchronized with the galvo-mirrors and only open when the femtosecond laser focus scanning in the region. The power of the femtosecond laser was about 8 mW measured at the specimen. The diameter of the laser focus point was approximately 0.7 μm. Images were recorded by confocal microscopy continuously before and after the stimulation. To take the real-time microscopy, the stimulation was set as a single frame of microscopy and inserted into the continuous confocal microscopy sequence at *t* = 5 s. In our experiments, each cell that was selected for UPLaS suffered only a single-time laser stimulation, during which only an individual mitochondrion was stimulated by the femtosecond laser.

### PINK1 knockout and Western blot

CRISPR sgRNA sequence was designed to target the PINK1 gene [NCBI Gene ID 65018] and sequence 3′-CCGGCCGGGCCTACGGCTTG GGG-5′ and 5′-GAAAGACTGCCCGGCCGCAA GGG-3′ in the exon 1 of the PINK1 precursor messenger RNA and 5′-CGTCTCGTGTCCAACGGGTC AGG-3′ in the exon 2 of the PINK1 precursor messenger RNA (GenBank accession NO.: NM_032409.3). The sgRNA sequences were used in conjunction with a Cas9-encoding plasmid, while HIV-1-U6-sgRNA-EFS-hspCas9-P2A-Puro was transformed from LentiCRISPRv2 (Addgene, Plasmid#98290) to generate PINK1-KO single-cell clones. The Puromycin-positive cells were sorted by flow cytometry and cloned. The effectiveness of knockout cells was verified before experiments.

PINK1 silencing efficiency was identified by Western blotting analysis. Cells were dissected in lysis buffer (RIPA: protease inhibitor mixture = 100:1) on the ice. Proteins were extracted by centrifuging for analysis in a western blotting experiment according to standard protocols. Proteins were separated on 4–12% BisTris NuPage gradient gels (Invitrogen) and transferred onto a Hybond‑P polyvinylidene difluoride membrane (Millipore). Membranes were blocked in 5% milk in TBS‑T for 2 h at room temperature, incubated with primary antibody (mouse anti-β-actin 1:5000, Sigma) (rabbit anti-PINK1 1:3000, Abcam) at 4 °C overnight, followed by incubation with secondary antibodies (rabbit anti-Flag 1:5000, Sigma) (mouse anti-Flag 1:5000, Sigma) for 1 h at room temperature. Proteins were visualized using the Western chemiluminescent HRP substrate (Millipore) on ECL enhanced chemiluminescence (Tanon 4200SF).

### Inhibition of mitochondria Ca^2+^ channel

For mitoROS scavenging, cells were treated with MitoTEMPO (SML0737, Sigma) for 1 h before experiments. For removal of cellular Ca^2+^, the cell medium was at first replaced with the Ca^2+^ free buffer for 15 min, followed by incubation with BAPTA-AM (10 μM) at 37 °C for 30 min. Cell Buffer was changed back to Ca^2+^ free medium for experiments. The Ca^2+^ free buffer was made up of 10 mM HEPES, and 20 μM EGTA (both from Sigma), and 10 mM glucose, 5 mM KCl, 140 mM NaCl, 1 mM MgCl_2_ (all from Sinopharm Chemical Reagent Co., Ltd.), and the pH value of the buffer was tuned to 7.4 by NaOH. For inhibition of mitochondria Ca^2+^ channels, Cyclosporine A (1 μM, Sigma) and Ruthenium red (10 μM, Abcam), and Ru360 (10 μM, Sigma) was used to treat cells for 30 min before detecting mitochondria Ca^2+^ or mitophagy by UPLaS.

### Assessment of Parkin and mitophagy initiation

To visualize intracellular Parkin, cells were transfected with Parkin-mCherry (Addgene, Plasmid#59419) and stained with MitoTracker Green (M36008, Invitrogen) simultaneously. For assessment of mitophagy, cells were transfected with GFP-LC3 (Addgene, Plasmid #22405) and simultaneously stained with MitoTracker Red (M22425, Invitrogen). Parkin recruitment and mitophagy initiation were, respectively, determined by the colocalization of mitochondria with Parkin and LC3-GFP. Carbonyl cyanide 3-chlorophenylhydrazone (CCCP, Sigma, 10 μM), HBSS (Gibco), and rotenone (Sigma, 10 μM) were used to treat cells for the indicated time in the main text and 24 h, respectively to induce mitophagy.

### PINK1 Immunofluorescence

Standard protocols from the manufacturers were used for immunofluorescence. The antibodies anti-PINK1 (1:500; Abcam) incubated with cells at 4 °C overnight, followed by incubation with secondary antibodies (rabbit anti-Flag 1:10000, Sigma).

### Statistical analyses

Statistical analysis was performed in GraphPad Prism 8 and all error bars displayed on graphs represent the mean ± SEM by the one-tailed paired *t-*test except stated otherwise. Plots of fluorescence intensity versus pixel were acquired by using the two-pixel-wide line-scan function of ImageJ software.

## Supplementary information

Supplementary figures

## Data Availability

The data that supports the findings of this study is available from the corresponding author upon reasonable request.
