# Supplementary material for: Mitochondrial Ca2+ oscillation induces mitophagy initiation through the PINK1-Parkin pathway
Source: Cell Death Dis. 2021 Jun 19;12(7):632. doi: 10.1038/s41419-021-03913-3 (PMC8214625; doi:10.1038/s41419-021-03913-3)
Supplement: Supplementary file 1 — Supplementary figures [file 41419_2021_3913_MOESM1_ESM.docx]

Supplementary figures

Fig. S1 The UPLaS induced selective autophagosomes fused with lysosomes.

Fig. S2 The DNA sequence of the PINK1-KO cell line.

Fig. S3 The MMP, mitoCa^2+^, and mitoROS responses to UPLaS in SH-SY5Y cells treated with Rotenone


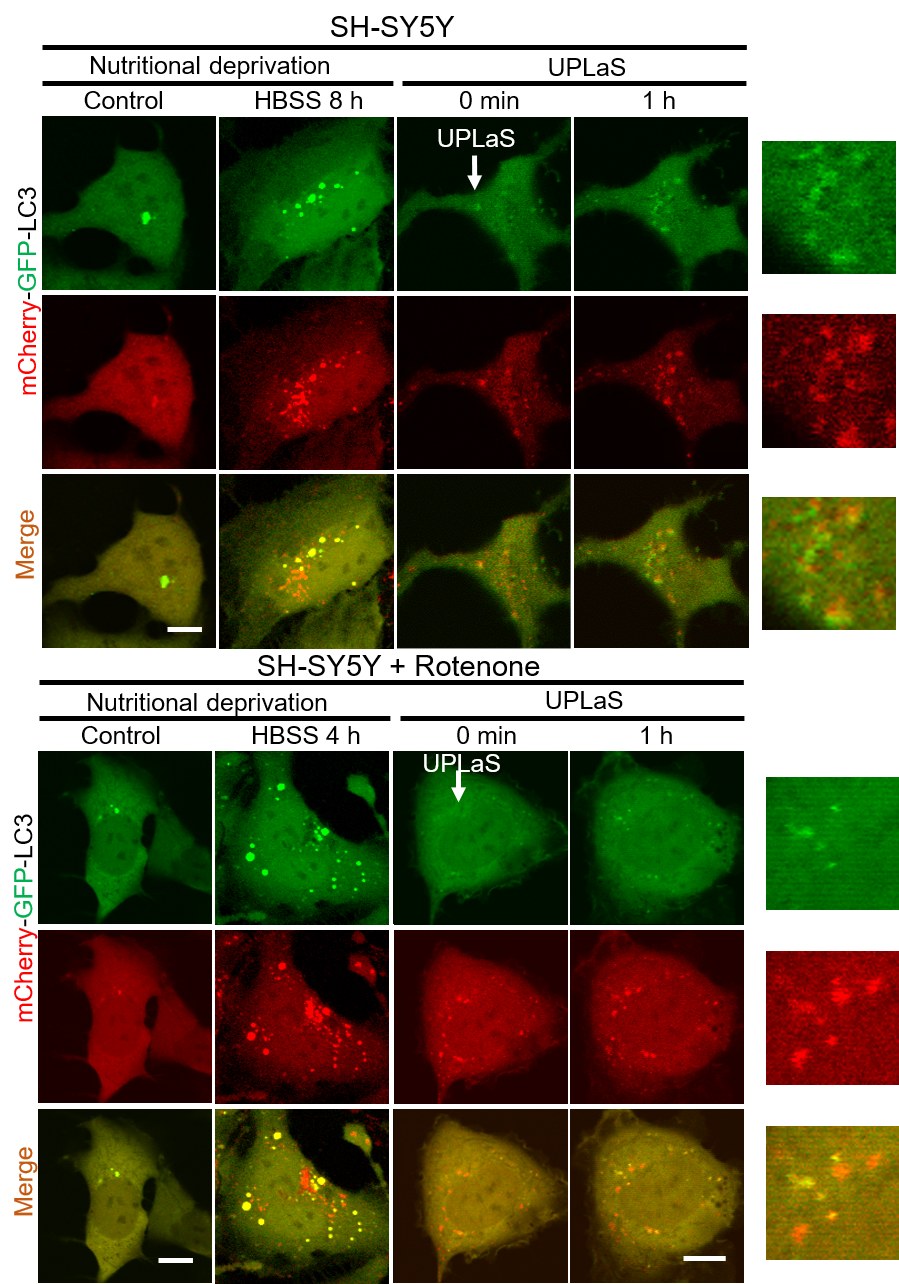


**Supplementary figure 1.** **The UPLaS induced autophagosomes fused with lysosomes.** The autophagosomes were indicated by mCherry and GFP. The autophagosomes fused with lysosomes were indicated by the quenched fluorescence of GFP since the low pH of the lysosomes would decrease the GFP fluorescence in the autophagosomes when they fused with lysosomes. The mCherry was not influenced. Positive control: cells treated with HBSS. Bar: 10 μm.


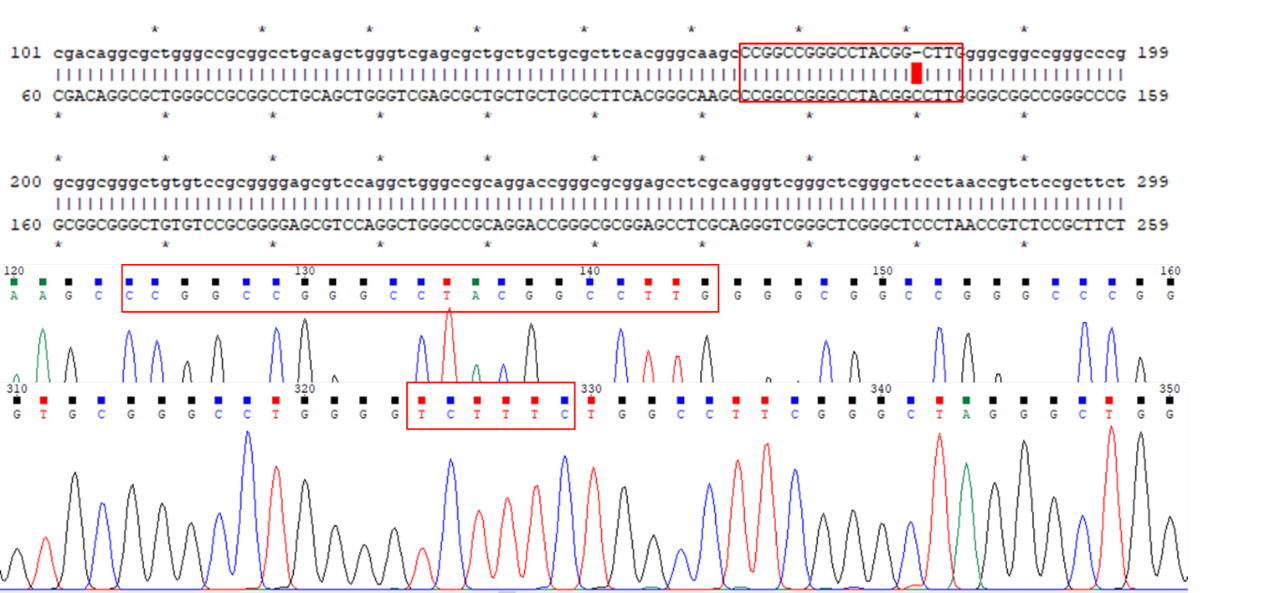


**Supplementary figure 2. The DNA sequencing map of the PINK1-KO cell line.** The map of DNA sequencing presented a single Peak. Compared with the wild type, a base C was inserted at the target FG-161 position, and a base (26 bp, not 3 times) was deleted at the target FG-162. A total of 25 bp size base sequence was deleted, indicating a PINK1 knockout homozygote.


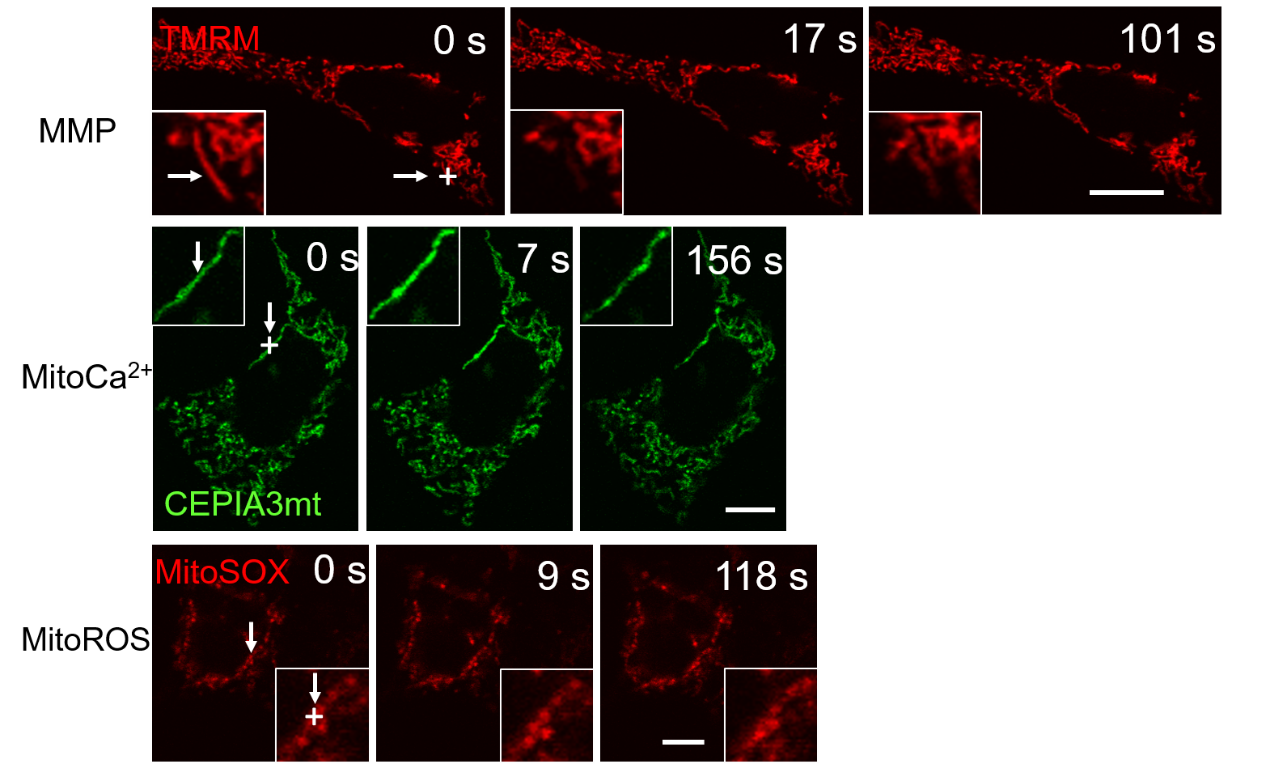


**Supplementary figure 3.** **The MMP, mitoCa^2+^, and mitoROS responses to UPLaS in SH-SY5Y cells treated with Rotenone.** Crosses: the location of UPLaS. Arrows: the target mitochondria. Inserts: magnifications of the target mitochondria. Bar: 10 μm.
